# Gene immunotherapy regulated by astrocytic reactivity in a mouse model of amyloidosis

**DOI:** 10.1002/alz70855_103554

**Published:** 2025-12-23

**Authors:** Chinaza Lilian Dibia, Nathalie Vacaresse, Rikke Han Kofoed, Brandy Laurette, Luis Fernando Rubio‐Atonal, Dildare Yurtsever, Isabelle Aubert

**Affiliations:** ^1^ University of Toronto, Toronto, ON, Canada; ^2^ Sunnybrook Research Institute, Toronto, ON, Canada; ^3^ Center for Experimental Neuroscience (CENSE), Department of Neurosurgery, Aarhus University Hospital, Aarhus N, Denmark, Aarhus N, Aarhus, Denmark; ^4^ University of Waterloo, Waterloo, ON, Canada

## Abstract

**Background:**

Recombinant adeno‐associated viruses (AAVs) capable of crossing the blood‐brain barrier (e.g. AAV.PHP.eB) and encoding antibodies against amyloid beta peptides (Aβ) have potential to evaluate brain‐wide gene immunotherapies in Alzheimer's disease (AD). Furthermore, leveraging astrocytic reactivity in response to Aβ pathology, the glial fibrillary acidic protein (GFAP) promoter could serve as a regulator of gene immunotherapy.

**Hypothesis:**

Reactive astrocytes can regulate the expression of the recombinant anti‐Aβ antibody (rSol) under the control of a GFAP promoter in the TgCRND8 (Tg) mouse model of amyloidosis.

**Method:**

To study GFAP expression in Tg mice, GFAP mRNA levels were quantified using qPCR in the hippocampal formation at 3, 5, and 6 months (*n* = 6 per group). Next, AAV.PHP.eB.GFAP.rSol‐myc‐tag and AAV.PHP.eB.GFAP.GFP were co‐injected intravenously in Tg mice while non‐Tg littermates and C57BL/6J mice served as controls. One‐month post‐injection, brain sections were processed for immunohistochemistry and RNAscope.

**Result:**

GFAP mRNA levels doubled in 6‐month‐old compared to 3‐month‐old Tg mice. Brain‐wide GFP expression in astrocytes confirmed efficacy of the GFAP promoter. Notably, brain cell transduction varied across Tg mice, peaking in the C57BL/6J line. Ly6A, a protein previously shown to facilitate AAV.PHP.eB entry into the brain, may explain this variability in transduction levels. We are currently examining Ly6A expression in our transgenic mouse line to determine the Tg background that will deliver the most efficient viral transduction.

**Conclusion:**

These results suggest that the GFAP promoter could control the production of therapeutics, such as rSol, in response to amyloid‐induced astrocytic reactivity. Long‐term studies will assess whether rSol prevents Aβ pathology progression in Tg‐Aβ mice.

